# *Elemental Home*: A Video Game to Explore Chemistry in Everyday Life

**DOI:** 10.1021/acs.jchemed.5c00168

**Published:** 2025-08-04

**Authors:** Pedro Juárez-González, María José Cano-Iglesias, Daniel Cebrián-Robles, Antonio Joaquín Franco-Mariscal

**Affiliations:** 152694 Universidad de Málaga, Science Education, Málaga 29071, Spain

**Keywords:** First-Year Undergraduate/General, High School/Introductory Chemistry, Physical Chemistry, Humor/Puzzles/Games, Periodicity/Periodic Table, Student-Centered Learning

## Abstract

This study presents and analyzes *Elemental Home*, a video game designed for teaching chemistry, with a specific focus on chemical elements in everyday life. Set inside a house, the video game situates learning in an everyday context, challenging players to identify chemical elements in household objects while reflecting on their environmental impact. This paper evaluates the learning potential and user experience of *Elemental Home*, based on the participation of 18 Spanish preservice chemistry teachers and 18 ninth-grade students. Learning in both groups was evaluated using data collected from the video game’s database, while usability and user satisfaction were assessed through a questionnaire. Additionally, ninth-grade students completed a pretest and post-test to measure their understanding of associations between chemical elements and everyday objects. Both students and preservice teachers surpassed 70% accuracy in element–object associations at level 1 (14/18 for students and 10/18 for preservice teachers), although students required more attempts on average to reach this level (4.22 compared to 2.28 attempts). While students progressed only to level 2, preservice teachers advanced as far as level 4. Additionally, *Elemental Home* delivers a positive user experience for preservice teachers (usability: 77.35/100; satisfaction: 75.00/100) and is regarded as moderately engaging by students (usability: 64.44/100; satisfaction: 64.50/100). Results emphasize the potential of video games in chemical education, demonstrating how the combination of game-based learning, contextualization, and interactive elements can significantly transform traditional teaching and learning approaches in chemistry.

## Graphical abstract

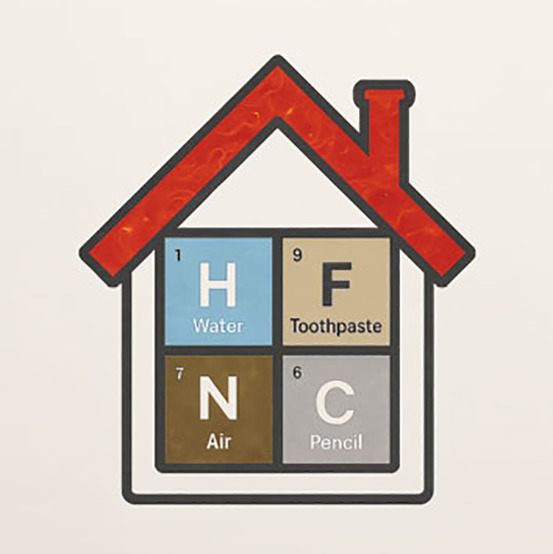

## Background

### Digital Transition

As technology advances, so do the methods by which people acquire, use, and share information.^1^ The COVID-19 pandemic brought about a drastic change in teaching and learning routines. This change created a complex and challenging situation that required rapid adaptation to new technologies in various educational aspects, such as assessment, content, and communication between students and teachers.^2^

Today, smartphones give students access to a virtual library’s worth of information, transforming how they learn.^3^ Smartphones have the potential to become powerful educational tools, fostering more interactive and meaningful learning.^4^ These devices allow for the creation of a dynamic space where students can practice and reinforce their chemistry knowledge through a wide variety of resources, such as educational video games, adventure games, simulations, interactive applications, and multimedia platforms.^5–10^ Many digital resources allow users to visualize chemical phenomena, facilitating the understanding of reactions, structures, and key processes in chemistry.^11^ Additionally, educational mobile apps offer a unique opportunity to engage students in environmental chemistry by providing interactive content that emphasizes sustainability.^12^ Furthermore, the ability to access knowledge anytime and anywhere fosters learning, strengthening student autonomy and intrinsic motivation.^13^ In this way, smartphones present new opportunities for enhancing chemistry education, as they not only provide immediate access to information but also promote active participation, critical thinking, problem-solving, and context-based learning, enriching the educational experience.^14^ In this context, games that connect chemistry to students’ everyday lives are relevant, as they can provide meaningful benefits to chemistry learning.^15^ Notable examples in the field of organic chemistry include games such as ChemPOV^16^ and Chem’Sc@pe.^17^

### Game-Based Learning in Chemistry Education

Game-based learning (GBL) applied to chemistry education, especially through mobile applications, is rapidly evolving.^4,18^ Games and video games effectively engage students with chemistry content in meaningful ways.^16^ Furthermore, when GBL in mobile applications is properly contextualized, it can become a powerful learning tool that facilitates active and constructive learning, making the experience more engaging and dynamic.^19,20^ The study by Ping et al.^21^ on game-based mobile chemistry applications concluded that increased use of these tools enhances student performance, particularly when they combine interactive tutorials with a guided learning approach. Additionally, a meta-analysis on the impact of digital games on learning revealed that these tools significantly improve learning from preschool to university compared to nongame-based approaches.^22^

In the context of the periodic table, chemistry teachers traditionally begin by presenting the table and its elements, followed by a memorization-based approach.^23^ However, this method is often perceived as repetitive and unengaging, which can negatively impact students’ motivation and interest in the subject.^24^ The literature review by Franco-Mariscal et al.^25,26^ on the use of games in teaching the periodic table identified two main types. The first type focuses on memorizing chemical names, symbols, and their positions in the periodic table using mnemonic strategies to aid retention, such as encoding, organization, and association. Examples include word-forming games and crosswords,^27^ drawings,^28^ card games,^29,30^ and songs.^31^ The second type emphasizes understanding and applying the periodic table. These games employ learning strategies that help students conceptualize, understand, and apply key aspects, fostering deeper reasoning and more meaningful learning. Examples include games that explore the environmental presence of elements through drawings in different contexts, such as a house,^32^ a car,^33^ or food;^34^ macroscopic properties using literary texts;^35^ atomic models through board games;^36^ atomic properties with bingo games;^37,38^ and periodicity using dominoes.^39^ In the past decade, the use of video games and mobile applications for teaching this topic has significantly increased.^10,40^

In this context, this paper presents and evaluates the video game *Elemental Home* as an educational tool for teaching the periodic table, contextualizing the learning of chemical elements through everyday objects and materials. It also seeks to assess the game’s usability and user satisfaction among preservice chemistry teachers and ninth-grade students.

## Video Game *Elemental Home*

### Description of the Video Game

*Elemental Home* is a single-player video game available in both Spanish and English, consisting of eight levels. After selecting an avatar, the video game begins with a tutorial that explains its mechanics and provides fundamental knowledge (Figure 1, left image). Players then complete an initial self-assessment to measure their ability to identify chemical elements present in their everyday environment. This test is repeated after reaching the fourth level and again at the end of the eighth level, allowing players to track their progress throughout the video game.

**Figure 1:**
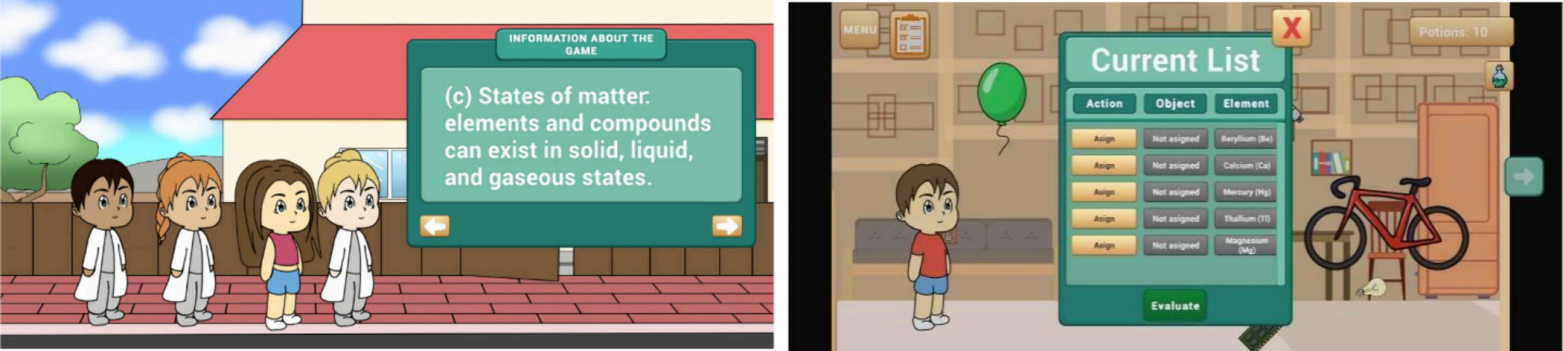
Tutorial (left image) and list of chemical elements to be assigned to objects (right image).

The video game includes 71 chemical elements, presented as elemental substances, chemical compounds, solutions, and alloys. Table 1 provides some examples. The selection included all elements of the periodic table, excluding those from the f-block (lanthanides and actinides), the last three elements of the sixth period, and the elements of the seventh-period with limited relevance to everyday contexts. Exceptions were made for elements with notable significance, such as uranium and plutonium. A complete list is available in Supporting Information A.

**Table 1:** Examples Illustrating How Chemical Elements Are Presented in the Video Game

Form of presentation	Example	Object
As an elemental substance	Carbon	Pencil lead
	Silver	Candlestick
	Tungsten	Light bulb filament
As a chemical compound	Fluorine (as sodium fluoride)	Toothpaste
	Lithium (as lithium oxide)	Batteries
	Gallium (as gallium arsenide)	Computer chip
As a solution	Chlorine and sodium (in an aqueous solution of sodium hypochlorite)	Bleach
	Nitrogen and hydrogen (in an aqueous solution of ammonia)	Ammonia
As an alloy	Niobium (alloyed with titanium)	Magnet
	Magnesium (alloyed with aluminum)	Racing bike frame
	Cerium and lanthanum (ferrocerium alloy)	Lighter flint

Players must assign chemical elements to the corresponding objects (Figure 1, right image) within various household settings. The same element may appear in different objects (e.g., nitrogen, as ammonia or fertilizer) and players can modify the assignment of an element by selecting a different associated object. As players progress through the levels, the number of chemical elements, scenarios, and objects all increase. Each level introduces a new scenario (level 1: living room and study; level 2: bathroom; level 3: kitchen; level 4: dining room; level 5: garage; level 6: garden; and level 7: terrace) while retaining those from previous levels. The level 8 differs in gameplay, requiring players to appropriately associate 20 objects within eight minutes to complete it. In terms of element placement within levels and scenarios, most elements appear randomly, while a select few are intentionally linked to specific contexts to enhance real-world relevance—for instance, uranium relates to the nuclear plant in the garden’s background.

The challenges presented in the game are set by a group of scientists, who also evaluate the players’ success. At the end of each level, players receive a report on the percentage of correctly identified chemical elements relative to the total, along with an assessment of their knowledge level (Figure 2, left image). Additionally, a leaderboard displays the highest scores, encouraging players to refine their learning and reflect on their progress.

**Figure 2:**
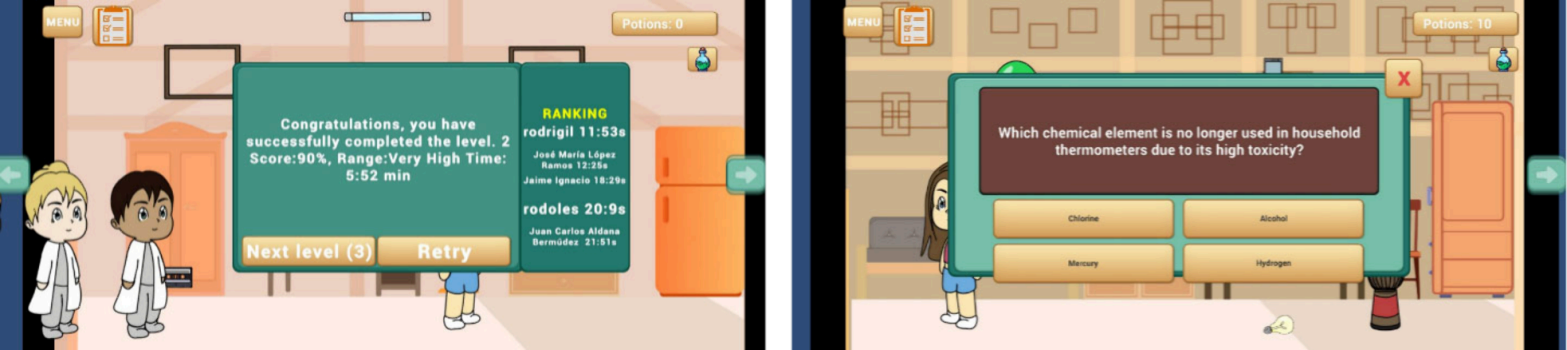
Level assessment and ranking (left image) and example of a question on ecological transition (right image).

As players explore the house, they can gather information about objects before assigning them to their list (e.g., ’Rat poisons contain barium as a chemical compound called barium carbonate’). To assist, the game provides 10 potions per level (top right corner of the screen). The feedback is important for reinforcing learning and also appears after the evaluation whether the association is correct or incorrect (e.g., ’The lanthanum-butane cylinder association is not valid. The gas in a butane cylinder is a chemical compound whose molecule contains four carbon atoms and ten hydrogen atoms’).

When a player runs out of potions, they can earn more by answering questions that encourage reflection on actions and decisions related to everyday objects and their chemical elements, fostering environmental care and responsible resource use (Figure 2, right image). Moreover, it aims to support ecological transition through chemistry education, raising awareness about environmental and health issues related to the use, management, and problems associated with various chemical elements.

Each question has four options; a correct answer lights the screen green, an incorrect one turns it red, giving immediate feedback. To avoid constant reliance on hints, the player must wait 20 seconds before requesting another one. An example of a question is ’In the 1950s, a petrochemical company working with mercury compounds was dumping its waste into the Minamata Bay in Japan. What consequences do you think these actions had?’ Possible answers include: ’In the following years, nearly 3,000 people were diagnosed with mercury-related diseases’; ’The fish and seafood from Minamata Bay tasted better’; ’A small environmental contamination occurred in the waters, which the bay itself was able to cleanse’; ’The fish and seafood from Minamata Bay were of higher quality.’

In summary, *Elemental Home* stands out for integrating several key features that differentiate it from other educational resources. First, the learning of chemical elements is contextualized in everyday life, specifically in relation to common objects, a context that has proven useful in nondigital formats to facilitate a closer and more meaningful understanding of chemistry.^32–34^ Second, *Elemental Home* not only promotes the digital transition in chemistry education by integrating video games into the classroom, but it also fosters the ecological transition. This is achieved by linking knowledge of chemical elements with environmental issues, encouraging reflection on the impact of these elements on the environment and the development of ecological awareness. In this way, the video game not only enriches the learning of chemistry but also contributes to a more sustainable education adapted to contemporary challenges. Finally, it is distinguished by its eight levels of learning, providing immediate feedback and including evaluation at selected levels. Additionally, its bilingual nature expands its accessibility.

### Technical Aspects of the Video Game

This video game was developed as part of the Spanish Government’s R&D project TED2021–130102B–I00, using the Unity engine, which employs OpenGL for Windows. This combination was chosen for its versatility, flexible development, and free licensing, ideal for noncommercial use. Unity’s powerful component-based system facilitates efficient game creation, while its robust support for 2D sprites and animations enables streamlined and optimized implementation. Additionally, OpenGL enables browser-based game distribution via WebGL, allowing Unity to render graphics without plugins.

The development environment for programming its components, functionalities, and mechanics was based on C# and PHP, the latter used for database connectivity. The avatars, scenarios, and objects were designed by a graphic designer using Sketchbook. The game is freely available on Android, Windows, and the web at https://encic.itch.io/elemental-home.

## Method

### Participants

This research included studies with Spanish preservice chemistry teachers and ninth-grade students. The sample was selected for convenience. No unexpected or unusually high safety hazards were encountered.

#### Study with Preservice Chemistry Teachers

The participants were 18 preservice chemistry teachers enrolled in a module on resources for teaching chemistry as part of the course *Teaching Innovation and Introduction to Educational Research* during the 2023–2024 year. This course is part of the Chemistry specialization in the Master’s Degree in Secondary Education Teaching at the University of Málaga (Spain). 72.2% were women, and 27.8% were men. The participants played the video game during a one-hour session.

Most preservice teachers (55.6%) had never used educational video games, while the rest had minimal experience either through training (38.9%) or leisure (5.5%). This limited exposure may affect their confidence in incorporating such tools into their teaching and highlights the need to promote digital transition in preservice teacher training programs.

The choice of preservice chemistry teachers was intentional, to provide opportunities to refine the design, functionality, and methodology of the video game before involving ninth-grade students. Feedback from preservice teachers led to several enhancements in the game: (a) the difficulty was increased by raising the level progression threshold from 60% to 80%, and (b) the interface and pacing were improved through better object placement and refined text content. This aims to improve the quality of the video game and ensure that its implementation with high school students will be more effective, as the resource will have been previously validated by preservice teachers.

#### Study with Ninth-Grade Students

The sample comprised 18 ninth-grade students, aged 15, with 55.6% identifying as female and 44.4% as male. Among them, 38.9% had never used educational video games, 50.0% had used educational apps during their studies, and 11.1% for leisure. These results indicate that ninth-grade students had slightly greater exposure than the preservice teachers. The participants played the video game during a one-hour session.

### Ethics Statement

The study was conducted in accordance with the protocol approved by the Ethics Committee on Experimentation of the University of Málaga (Spain) (CEUMA) with reference number 126–2023-H. The formal procedures followed included obtaining informed consent from the participants, with the option to decline or with draw participation.

### Data Collection and Analysis

Three instruments were used for data collection: the database associated with the video game, a questionnaire on user satisfaction and game usability (administered to both preservice teachers and students), and a pre/post-test to assess knowledge of the relationship between chemical elements and daily life, which was applied only to students.

The correct answers stored in the database for each player served as an indicator of learning at each level. For analysis, the category system used by the game for feedback and level progression was applied to assess the learning: very low (0–24%), low (25–49%), medium (50%–69%), high (70–89%), and very high (90–100%).

Regarding usability and satisfaction, a validated questionnaire developed by Serrano and Cebrián-Robles^41^ was administered online after playing the video game. This questionnaire consists of 26 items on a 5-point Likert scale (from ‘strongly disagree’ to ‘strongly agree’) and evaluates aspects such as ease of use, satisfaction, technical issues, graphic design, and overall user experience. To limit impulsive responses, the instrument combines direct (5 = best) and indirect (1 = best) items.^42^ The questionnaire demonstrated strong reliability (Cronbach’s alpha = 0.889)^38^ and was refined through three iterations to be adaptable to different educational tools.^41^ The questions were grouped into two analysis blocks: usability (items Q1 to Q10) (Table 2) and satisfaction (items Q11 to Q26) (Table 3). The analysis was conducted by calculating an average score for each block on a 0 to 100 scale. Additionally, the mean, median, and standard deviation were calculated directly for all items.

**Table 2:** Usability Findings

	**Preservice teachers**				**Students**		
**Usability item**	**Character**	**x̅**	**Median**	**σ**	**x̅**	**Median**	**σ**
Q1. The video game was easy to use.	Direct	4.47	5	0.62	3.94	4.5	1.35
Q2. Incompatibilities appeared that made it difficult for me to handle.	Indirect	3.53	3	1.23	3.33	3	1.28
Q3. It can be used without prior explanations.	Direct	3.59	4	1.18	3.61	4	1.29
Q4. I found the editing complicated.	Indirect	4.06	4	0.89	3.78	4	1.31
Q5. I got disoriented at some point with the video game.	Indirect	4.00	4	0.87	2.94	3	1.39
Q6. The menu options are clear.	Direct	4.24	4	0.75	4.00	4	0.97
Q7. I needed little time to manage the video game.	Direct	4.06	4	1.20	3.89	4	1.28
Q8. I needed help to access it.	Indirect	4.00	4	1.06	3.06	3	1.39
Q9. I encountered technical problems.	Indirect	3.53	4	1.37	3.67	4	1.53
Q10. At some point I felt panic.	Indirect	4.88	5	0.48	3.56	5	1.76

**Table 3:** Satisfaction Findings

	**Preservice teachers**				**Students**		
**Satisfaction item**	**Character**	**x̅**	**Median**	**σ**	**x̅**	**Median**	**σ**
Q11. The video game was pleasant to use.	Direct	4.06	4	0.66	3.78	4	1.06
Q12. Using the video game was exhausting.	Indirect	3.94	4	1.01	3.50	4	1.46
Q13. I found working with the video game motivating.	Direct	3.94	4	0.83	3.72	4	0.89
Q14. I would have preferred to use another familiar video game instead of this one.	Indirect	4.53	5	0.62	3.61	4.	1.50
Q15. The options in the video game were as expected.	Direct	4.18	4	0.73	3.39	3	1.42
Q16. It was very laborious to do something with the video game.	Indirect	4.47	5	0.62	3.06	3	1.51
Q17. I would use the video game again if needed.	Direct	4.00	4	0.87	3.67	4	1.50
Q18. I found some options difficult to interpret.	Indirect	3.65	4	1.22	3.11	3	1.45
Q19. It requires help from an expert.	Indirect	4.65	5	0.70	3.72	4.5	1.67
Q20. The graphic design is poor.	Indirect	3.35	3	1.22	3.56	4	1.58
Q21. I would recommend the video game to others.	Direct	4.06	4	0.75	3.61	4	1.30
Q22. The response time during interaction is slow.	Indirect	4.06	4	1.03	3.78	4.5	1.48
Q23. The responses given are hard to understand.	Indirect	4.35	5	0.86	2.89	3	1.18
Q24. The help to understand the video game was useful.	Direct	4.35	4	0.79	3.83	4	1.10
Q25. The editing is very flexible.	Direct	3.47	3	0.94	3.94	4	0.87
Q26. Overall, I am satisfied with the video game.	Direct	4.00	4	0.71	4.11	4.5	1.08

The questionnaire also included an additional item specific to each group. Preservice teachers were asked: *Would you use Elemental Home in your future* *teaching practice?* to assess the potential implementation of the video game in educational settings. Ninth-grade students responded to the question: *Would you like to use the Elemental Home outside of class?* Both items were answered using a dichotomous scale (yes/no).

The pre/post-test consisted of listing as many chemical elements as the students knew, along with corresponding everyday objects or materials associated with each element. For each student, the number of appropriate element/object associations was recorded at both points, and the Wilcoxon test was used to identify any statistically significant differences. The tests were administered online, outside the game, as few students were expected to reach level 4, where this activity is included.

## Results

### Learning Achieved

#### Study with Preservice Chemistry Teachers

Figure 3 displays preservice chemistry teachers’ achievement at each level, based on learning outcomes measured through in-game performance data. During the play session, a significant number of participants played each level multiple times, repeating it until they achieved at least 60% correct answers to progress to the next level. The Figure 3 includes all players for level 1, while for subsequent levels, only those who successfully completed them are represented. If the preservice teacher plays multiple games at the same level to surpass it, the game with the highest score will be displayed. Average attempts per level were: 2.28 (level 1), 1.47 (level 2), 1.09 (level 3), and 1.00 (level 4).

**Figure 3:**
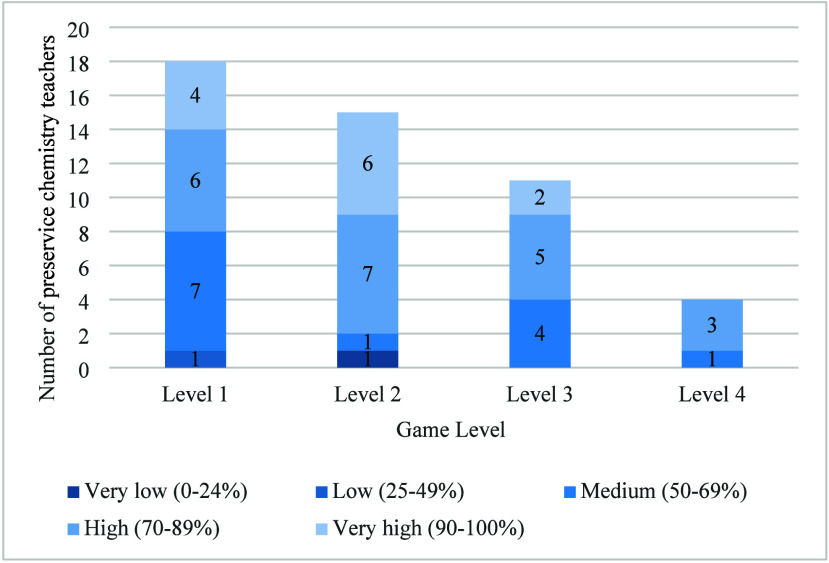
Distribution of preservice chemistry teachers according to their level of learning.

After one hour, participants reach only level 4 of 8, indicating slow game progress and a significant time investment needed, even for university-level chemistry players, reflecting the learning curve and challenge.

In level 1, where players search for 5 elements among 14 objects across two scenarios, most preservice teachers achieve a high (6/18) or very high (4/18) performance, suggesting this level is relatively accessible. However, 7/18 show medium performance, likely reflecting their ongoing adjustment to the game mechanics.

Level 2 involves searching for 10 elements among 19 objects across three scenarios. The findings are similar to those in level 1, with a strong presence of players with high (7/15) and very high (6/15) performance. Nevertheless, there is a decrease in the players with medium performance (1/15), suggesting that most preservice teachers have already internalized the game’s mechanics.

There is a notable decrease in the total of preservice teachers (11) reaching level 3. Among them, the proportions of medium (4/11) and high (5/11) performance increase, while very high performance declines (2/11). This reflects the significant difficulty jump at level 3, which involves locating 15 elements across four scenarios with 27 objects, making it challenging for some players to achieve maximum performance.

Finally, level 4 only presents players in the medium (1/4) and high (3/4) categories. The absence of players with very high performance could indicate that this level, which involves searching for 20 elements in five scenarios among 36 available objects, is the most difficult of all, as no participant reaches the 90–100% learning range. Moreover, the limited participation of preservice teachers in the higher levels is explained by the considerable time spent on the initial levels, which hindered their progress, as the total game time was limited to one hour.

#### Study with Ninth-Grade Students

Figure 4 shows the achievement reached by the ninth-grade students at each level.

**Figure 4:**
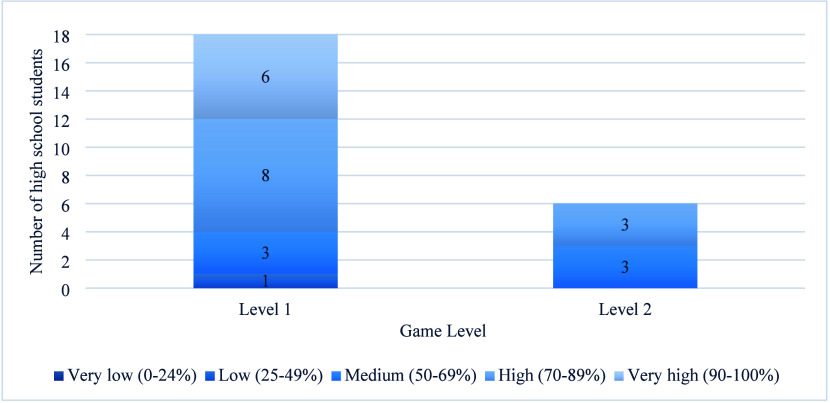
Distribution of ninth-grade students according to their level of learning.

The main difference between the preservice teachers and the ninth-grade students was that the latter only reached level 2 after one hour of gameplay. This outcome may be attributed to the increase in the progression threshold to 80%, as recommended by the preservice teachers, with the aim of reinforcing students’ learning before introducing new content. Level 1 proved more challenging for the students, who required an average of 4.22 attempts to complete it, whereas preservice teachers needed only 2.28. However, this extended engagement contributed to stronger overall performance, as anticipated by the preservice teachers, with 8/18 students reaching a high level of achievement and 6/18 achieving very high performance. The remaining 4 students scored at medium (3/18) or low (1/18) performance.

Among the students who reached level 2, only six successfully completed it—three with high and three with medium performance—averaging 1.67 attempts. Although they initiated level 3, none were able to complete it within the session time.

The average element–object associations increased from 1.56 before to 2.78 after playing the game. The Wilcoxon test revealed a statistically significant improvement favoring the post-test (Z = −2.412; p = 0.016). Specifically, 11/18 students showed improved results, five remained unchanged, and only two experienced a decrease in their scores. These findings suggest that the intervention had a positive effect on most participants, supporting the educational value and effectiveness of the video game.

### Usability of the Video Game

Table 2 shows the mean, median, and standard deviation for each item in the usability dimension for preservice teachers and students. Usability scores averaged 77.35/100 for preservice teachers and 64.44/100 for ninth-grade students. While scores above 75 reflect a positive perception of ease of use—achieved only by preservice teachers—students’ moderate score points to areas for improvement, but also highlights the game’s potential to lower technological barriers in educational settings.

The high rating given by preservice teachers for the item *The video game was easy to use* (Q1: x̅ = 4.47) and their very low perception of panic (Q10: x̅ = 4.88, indirect item) suggest that the game’s design successfully minimizes initial friction and technological anxiety, two critical factors for the effective adoption of educational tools.^43^ In contrast, students reported lower ease of use (Q1: x̅ = 3.94) and higher perceived panic (Q10: x̅ = 3.56), suggesting that unfamiliarity with the subject may heighten anxiety during gameplay. Nonetheless, more moderate responses to the item *It can be used without prior explanations* (Q3: x̅ = 3.59 for preservice teachers, x̅ = 3.61 for students), along with ratings related to technical issues (Q9: x̅ = 3.53 versus 3.67) and compatibility problems (Q2: x̅ = 3.53 versus 3.33) highlight areas for improvement. As noted by Bui et al.,^44^ enhancing game usability and improving the clarity of the user interface can significantly improve participants’ overall gaming experience. For *Elemental Home*, refining the introductory tutorial and optimizing error handling may strengthen user autonomy and reduce potential frustration, ultimately supporting a smoother and more engaging learning process.

### Satisfaction with the Video Game

The satisfaction dimension, which assesses the overall experience with the video game, showed an average score of 75.00 for preservice teachers and 64.50 for students. This score fell below the 75-point threshold for the students, indicating that their experience was less enjoyable and motivating compared to that of the preservice teachers. This is particularly significant in GBL, where maintaining student engagement and fostering a positive attitude toward learning chemistry are crucial. Several factors may have influenced these findings, such as the fact that more students than preservice teachers had to replay level 1 several times to advance, or the differing expectations between the two groups—preservice teachers may have focused more on the educational potential of the game, while students may have been more interested in its entertainment value. Table 3 presents the mean, median, and standard deviation for each item in the satisfaction dimension.

Although preservice teachers generally achieved higher scores than students, both groups rated the user experience as positive finding it enjoyable (Q11: x̅ = 4.06 for preservice teachers; 3.78 for students) and expressing overall satisfaction with the game (Q26: x̅ = 4.00 vs 4.11).

According to Felício and Soares,^45^ the capacity of an educational game to foster learner autonomy aligned with instructional goals is a critical success factor. In this regard, the findings showed higher autonomy among preservice teachers (Q19: x̅ = 4.65, indirect item) compared to students (x̅ = 3.72). This contrast suggests that students may require additional guidance and scaffolding to engage confidently with the video game, potentially due to their lower familiarity with the chemical knowledge addressed in *Elemental Home*.

Additional differences emerged between the two groups regarding their preference for this video game compared to other options. Preservice teachers found the experience notably distinctive (Q14: x̅=4.53, indirect item), while students were less convinced (x̅ = 3.61), suggesting it stands out less within their digital experiences.

Indicators such as motivation (Q13: x̅ = 3.94 for preservice teachers; 3.72 for students), willingness to recommend the game (Q21: x̅ = 4.06 vs 3.61), and intention to use it in the future (Q17: x̅ = 4.00 vs 3.67) highlight the video game’s potential for fostering engagement, an essential component in GBL.^46–48^

However, both groups rated the graphic design modestly (Q20: x̅ = 3.35 for preservice teachers; 3.56 for students, indirect item), indicating an opportunity for improvement. Since visual appeal enhances immersion and enjoyment^44^, improving aesthetics could boost satisfaction and the game’s educational impact.

### Video Game Potential for Formal and Informal Learning Contexts

All preservice teachers expressed their intent to use *Elemental Home* in their future teaching practice, perceiving it as useful, engaging, and supportive of chemistry learning. By contextualizing chemistry to everyday materials, the game highlights its practical relevance and promotes active, autonomous learning through exploration and feedback. Additionally, its game-based format also boosts motivation and engagement with the chemistry content.

Despite their limited prior experience with video games, preservice teachers view *Elemental Home* as an effective resource for chemistry education. This positive assessment could facilitate a broader adoption of the game in their future professional settings, contributing to the integration of technologies in the chemistry classroom.

The game’s educational value extended beyond the classroom, with 77.77% of students interested in using it informally.

## Conclusions

The findings highlight the transformative potential of video games in chemistry education,^15–17^ particularly through *Elemental Home* in preservice teacher training and ninth-grade students.

High learning performance in linking chemical elements to everyday life was observed up to level 4 for preservice teachers and level 2 for students. Additionally, usability (preservice teachers: 77.35; students: 64.44) and satisfaction (preservice teachers: 75.00; students: 64.50) received good ratings, which is significant given that 55.6% of preservice teachers and 38.9% of students had no prior experience with educational video games, demonstrating their ability to overcome initial technological barriers.

These findings reflect a positive attitude toward integrating technology into chemistry teaching and a clear interest in incorporating these resources into teaching practice. The favorable evaluation of *Elemental Home* aligns with previous research emphasizing the effectiveness of contextualizing chemical elements in everyday settings^32–34^ and studies that demonstrate the impact of digital games on learning.^22^

The evaluation by preservice teachers suggests that *Elemental Home* is not only a viable tool, but also exhibits key qualities for effective educational integration. The positive usability ratings suggest low operational complexity, allowing players to focus on learning chemistry through everyday contexts. Moreover, the satisfaction scores reflect that the experience was perceived as both enjoyable and motivating, two essential factors in GBL, as they can promote voluntary engagement and foster a more positive disposition toward chemistry.

The game’s functionality stands out for its intuitive interface, clear options, and efficient feedback delivery. However, areas for improvement were identified, such as the number of levels, graphic design, editing flexibility, and tutorial instructions. Optimizing these elements is key to maximizing its educational impact and enhancing the user experience.

Finally, this study underscores the potential of *Elemental Home* as a digital transition tool focused on the periodic table. All preservice teachers expressed their intent to implement it in their practice, assigning a recommendation score of 4.06/5. Additionally, 77.77% of the students reported that they would like to use the video game outside the classroom, emphasizing its appeal in informal learning contexts. These findings suggest that, with proper support and training, video games can be effectively integrated into educational settings, offering engaging methods for teaching chemistry and fostering environmental awareness.

## Educational Implications

This study highlights the need to promote the video games in chemistry education and strengthen digital competences in teacher training. Integrating these resources into the classroom not only enhances instruction but also facilitates learning in an increasingly digitalized educational landscape.

The video game’s good usability and satisfaction, combined with preservice teachers’ willingness to implement it, indicate a promising path forward. However, its adoption requires a gradual and systematic approach, starting with guided sessions to ensure effective adaptation. The video game’s flexibility allows its use across different educational levels, while its bilingual nature and progressive support system enable personalized learning experiences. Another notable feature is the integration of sustainability through questions on environmental impact, linking chemistry to real-world challenges for more contextualized learning.

For effective implementation, *Elemental Home* should complement—rather than replace—traditional methods within broader teaching-learning sequences. Additionally, training and support are recommended for preservice teachers with limited gaming experience.

## Study Limitations and Future Directions

One of the main limitations of the video game is the significant increase in complexity from level 3 onward, due to the large number of chemical elements that players must identify. This challenge, more evident in students, may enhance the learning experience, but could also introduce difficulties that increase cognitive load and potentially impact performance and satisfaction. To address this limitation, future versions of the video game will aim to reduce the time required to progress between levels, either by decreasing the number of elements or by introducing collaborative gameplay.

Another limitation lies in the game’s simple mechanics, centered solely on identifying chemical elements in everyday objects. Future versions could incorporate problem-solving tasks and collaborative challenges, more complex gameplay elements that can foster deeper cognitive engagement and higher-order thinking skills.

One last limitation is the sample size. Future research should focus on conducting longitudinal studies with larger samples to evaluate the long-term impact on learning, particularly among ninth-grade students.

The following improvements are planned for future updates of the video game: (a) adding explanations of lesser-known chemical elements to the tutorial to enhance player experience and reduce frustration with unfamiliar content; (b) providing feedback on ecological transition questions turning incorrect answers into learning opportunities; (c) relocating the self-assessment to the end of level 1 to ensure all players have the chance to complete it; (d) promoting inclusion and diversity by introducing avatars with varied skin tones and customizable features, for a more respectful and representative experience; (e) enhancing the leaderboard by incorporating both completion time and accuracy percentage, providing a more comprehensive assessment of student performance; and (f) unlocking all levels to allow players to access any level directly without restarting the game in each session.

Finally, developing new video games to further enrich chemistry education—especially in the context of ecological transition, highlighting the potential of the games to help transform society toward more sustainable environmental models—represents a promising direction for future work.

## Supplementary Material









## Notes

List of the chemical elements featured in the video game (PDF, DOCX)
User manual for how to download, install, and play the video game (PDF, DOCX)
