# Supplementary material for: Elemental Home: A Video Game to Explore Chemistry in Everyday Life
Source: J Chem Educ. 2025 Aug 4;102(8):3716–24. doi: 10.1021/acs.jchemed.5c00168 (PMC12355905; doi:10.1021/acs.jchemed.5c00168)
Supplement: Supplementary file 1 [file ed5c00168_si_001.pdf]

SUPPORTING INFORMATION A

Elemental Home: A video game to explore chemistry in everyday life

Pedro Juárez-González*, María José Cano-Iglesias, Daniel Cebrián-Robles, and Antonio Joaquín Franco-Mariscal

Universidad de Málaga, Science Education, 29010 Málaga, Spain. Email: pedrojg94@uma.es

List of the chemical elements featured in the video game

Z	ELEMENT	OBJECT	DESCRIPTION
1	Hydrogen	Water bottle	Water is a chemical compound whose molecule is formed by two hydrogen atoms and one oxygen atom.
1	Hydrogen	Ammonia	Ammonia is a chemical compound whose molecule is formed by one nitrogen atom and three hydrogen atoms.
1	Hydrogen	Butane cylinder	Butane in a cylinder is a chemical compound whose molecule contains four carbon atoms and ten hydrogen atoms.
2	Helium	Balloon	The gas in the fairground balloons is helium.
3	Lithium	Batteries	Batteries contain lithium in the form of compounds such as lithium cobalt oxide or lithium iron phosphate.
4	Beryllium	Wristwatch	Some wristwatches use beryllium alloys.
5	Boron	Tennis racket	Some tennis rackets are made of boron because it is a very light and stiff material.
6	Carbon	Butane cylinder	Butane in a cylinder is a chemical compound whose molecule contains four carbon atoms and ten hydrogen atoms.
6	Carbon	Pencil	The lead of a pencil is made of carbon in the form of graphite.
7	Nitrogen	Ammonia	Ammonia is a chemical compound whose molecule is formed by one nitrogen atom and three hydrogen atoms.
7	Nitrogen	Fertilizer	Fertilizers used in agriculture provide nitrogen by including urea, ammonium nitrate or ammonium sulfate in their composition.
8	Oxygen	Water bottle	Water is a chemical compound whose molecule is formed by two hydrogen atoms and one oxygen atom.
9	Fluorine	Toothpaste	Toothpaste contains fluoride and sodium in the form of sodium fluoride to strengthen enamel.
10	Neon	Car headlight	The gas in a car's headlights is neon.
11	Sodium	Bleach	Bleach is a solution containing chlorine and sodium in the form of sodium hypochlorite.
11	Sodium	Toothpaste	Toothpaste contains fluoride and sodium in the form of sodium fluoride to strengthen enamel.
11	Sodium	Salt	Table salt, or sodium chloride, is a chemical compound consisting of sodium and chlorine.
12	Magnesium	Racing bikes	The frames of some racing bikes are made of magnesium alloys.
13	Aluminium	Italian coffee maker	Italian coffee makers are made of aluminium alloys.
13	Aluminium	Window frame	A window frame is made of aluminium alloy.
13	Aluminium	Rocket	Some components in the aerospace industry are made of aluminium and scandium alloys.
13	Aluminium	Fork	Forks are usually made of aluminium.

14	Silicon	Window glass	Window glass includes silicon dioxide among its components.
14	Silicon	Camera lens	The lens of a camera contains silicon.
14	Silicon	Credit card	The microchip in credit cards is made of antimony on crystalline silicon.
15	Phosphorus	Matches	Matches contain phosphorus.
16	Sulfur	Anti-dandruff shampoo	Anti-dandruff shampoo contains selenium disulphide, combining sulfur for its purifying benefits with selenium for its antifungal properties.
17	Chlorine	Bleach	Bleach is a solution containing chlorine and sodium in the form of sodium hypochlorite.
17	Chlorine	Salt	Table salt or sodium chloride is a chemical compound consisting of sodium and chlorine.
18	Argon	Fluorescent lamp	The gas contained in an energy-saving fluorescent lamp is a mixture of argon and krypton, which reduces energy consumption.
19	Potassium	Banana	Bananas are rich in potassium.
20	Calcium	Milk	Milk is a good source of calcium.
21	Scandium	Rocket	Some components in the aerospace industry are made of aluminium and scandium alloys.
22	Titanium	Supermagnetic magnet	Some types of magnets are made of niobium and titanium alloys.
22	Titanium	Nail polish	Nail polishes are made of titanium dioxide.
22	Titanium	Painting	Paints use titanium dioxide as a white pigment.
23	Vanadium	Adjustable wrenches	Adjustable wrenches often contain vanadium in their steel to enhance strength and durability."
24	Chrome	Audio tape	A magnetic audio tape consists of a layer of chromium dioxide on a layer of diiron trioxide.
25	Manganese	Safe deposit box	Safes are made of a manganese steel alloy.
26	Iron	Audio tape	A magnetic audio tape consists of a layer of chromium dioxide on a layer of diiron trioxide.
27	Cobalt	Razor blade	Some razor blades are made of cobalt steel alloys.
28	Nickel	Euro coin	€1 and €2 coins are made from metal alloys that include nickel, such as cupronickel and nickel brass, to enhance durability.
29	Copper	Solar panel	Some solar panels are made using copper indium selenide, a material used in thin-film photovoltaic technology.
30	Zinc	Anti-corrosion coating	Some materials are coated with zinc as a protective layer against steel corrosion.
31	Gallium	Computer memory	Many computer components are made of arsenic and gallium in the form of gallium arsenide.
32	Germanium	Night vision goggles	The lenses of night vision or infrared goggles are made of germanium.
33	Arsenic	Computer memory	Many computer components are made of arsenic and gallium in the form of gallium arsenide.
33	Arsenic	Buckshot	Pellets are small lead spheres, to which arsenic is added to harden the lead.
34	Selenium	Anti-dandruff shampoo	Anti-dandruff shampoo contains selenium disulfide, combining sulfur for its purifying benefits with selenium for its antifungal properties.
35	Bromine	Photographic film	Photographic films contain bromine and silver as they are made by a layer of silver bromide on cellulose acetate.
36	Krypton	Fluorescent lamp	The gas contained in an energy-saving fluorescent lamp is a mixture of argon and krypton, which reduces energy consumption.
37	Rubidium	Purple fireworks	The purple color of fireworks is due to rubidium present as rubidium salts.
38	Strontium	Red fireworks	The red color of fireworks is due to strontium in the form of strontium nitrate or strontium carbonate.
39	Yttrium	Television screens	Yttrium oxide is used in television screens to generate red light in color displays.

40	Zirconium	Capsule for percussion instrument	The capsules of some percussion musical instruments are made of zirconium alloys.
41	Niobium	Supermagnetic magnet	Some types of magnets are made of niobium and titanium alloys.
42	Molybdenum	Lubricant	Some lubricants are made of molybdenum disulfide.
43	Technetium	Test tube with radioactive material	The test tube stored in the medical coat contains technetium, a radioisotope used in nuclear medicine for disease diagnosis.
44	Ruthenium	Fountain pen nib	Some ruthenium and osmium compounds are used to manufacture fountain pens.
45	Rhodium	Exhaust pipe	The catalytic converters used by automobiles in the exhaust pipe to reduce pollution from combustion gases are made of rhodium, platinum or palladium.
46	Palladium	Exhaust pipe	The catalytic converters used by automobiles in the exhaust pipe to reduce pollution from combustion gases are made of rhodium, platinum or palladium.
47	Silver	Candlestick	The candlesticks are made of silver.
47	Silver	Photographic film	Photographic films contain bromine and silver as they are made by a layer of silver bromide on cellulose acetate.
48	Cadmium	Screw	Some screws are made of cadmium alloys.
49	Indium	Solar panel	Some solar panels are made using copper indium selenide, a material used in thin-film photovoltaic technology.
50	Tin	Canned food can	The tinplate of canned food containers has a tin coating to protect it.
51	Antimony	Credit card	The microchip in credit cards is made of antimony on crystalline silicon.
52	Tellurium	Rubber	The vulcanization of car tire rubber uses tellurium.
53	Iodine	Shrimp	Shrimp are rich in iodine.
53	Iodine	Halogen lamp	Halogen lamps contains iodine.
54	Xenon	Projector	Xenon gas is used in projection lamps as a light source.
55	Cesium	Elevator	Infrared sensors in elevator doors use cesium nitrate.
56	Barium	Rat poison	Rat poison includes a chemical compound called barium carbonate.
57	Lanthanum	Lighter flints	Lighter flints are made of ferrocerium alloy, which contains cerium and lanthanum as the main elements.
72	Hafnium	Nuclear submarine	Some nuclear submarines use hafnium in the nuclear reactor control rods.
73	Tantalum	Mobile phones	Mobile phones contain tantalum, mainly in the form of tantalum capacitors used for efficient energy storage.
74	Tungsten	Light bulb filament	The filament of an incandescent bulb is made of tungsten.
74	Tungsten	Gas kitchen	The thermocouple used by a gas kitchen oven to measure temperature is made of tungsten and rhenium.
75	Rhenium	Gas kitchen	The thermocouple used by a gas kitchen oven to measure temperature is made of tungsten and rhenium.
76	Osmium	Fountain pen nib	Some ruthenium and osmium compounds are used to manufacture fountain pens.
77	Iridium	Spark plug	Car spark plugs are made of an iridium alloy to facilitate cold ignition.
78	Platinum	Exhaust pipe	The catalytic converters used by automobiles in the exhaust pipe to reduce pollution from combustion gases are made of rhodium, platinum or palladium.
79	Gold	Medal	The medals awarded at the Olympics are often made of gold.
80	Mercury	Thermometer	Several decades ago, thermometers used mercury as a liquid that expands or contracts with temperature changes. They are no longer sold because of their toxicity.
81	Thallium	Insecticide	Some insecticides contain thallium, but its use has been discontinued in some countries due to its high toxicity.
82	Lead	Car battery	A car battery is made up of a container with several lead plates submerged in sulfuric acid.

82	Lead	Buckshot	Pellets are small lead spheres, to which arsenic is added to harden the lead.
83	Bismuth	Fire extinguisher	Fire extinguishers are made from alloys containing bismuth.
92	Uranium	Nuclear fuel	Nuclear power plants use uranium and plutonium as fuel.
94	Plutonium	Nuclear fuel	Nuclear power plants use uranium and plutonium as fuel.
