# Supplementary material for: Elemental Home: A Video Game to Explore Chemistry in Everyday Life
Source: J Chem Educ. 2025 Aug 4;102(8):3716–24. doi: 10.1021/acs.jchemed.5c00168 (PMC12355905; doi:10.1021/acs.jchemed.5c00168)
Supplement: Supplementary file 2 [file ed5c00168_si_002.docx]

**SUPPORTING INFORMATION A**

***Elemental Home*: A video game to explore chemistry in everyday life**

Pedro Juárez-González*, María José Cano-Iglesias, Daniel Cebrián-Robles, and Antonio Joaquín Franco-Mariscal

Universidad de Málaga, Science Education, 29010 Málaga, Spain. Email: pedrojg94@uma.es

**List of the chemical elements featured in the video game**

| Z | ELEMENT | OBJECT | DESCRIPTION |
| --- | --- | --- | --- |
| 1 | Hydrogen | Water bottle | Water is a chemical compound whose molecule is formed by two hydrogen atoms and one oxygen atom. |
| 1 | Hydrogen | Ammonia | Ammonia is a chemical compound whose molecule is formed by one nitrogen atom and three hydrogen atoms. |
| 1 | Hydrogen | Butane cylinder | Butane in a cylinder is a chemical compound whose molecule contains four carbon atoms and ten hydrogen atoms. |
| 2 | Helium | Balloon | The gas in the fairground balloons is helium. |
| 3 | Lithium | Batteries | Batteries contain lithium in the form of compounds such as lithium cobalt oxide or lithium iron phosphate. |
| 4 | Beryllium | Wristwatch | Some wristwatches use beryllium alloys. |
| 5 | Boron | Tennis racket | Some tennis rackets are made of boron because it is a very light and stiff material. |
| 6 | Carbon | Butane cylinder | Butane in a cylinder is a chemical compound whose molecule contains four carbon atoms and ten hydrogen atoms. |
| 6 | Carbon | Pencil | The lead of a pencil is made of carbon in the form of graphite. |
| 7 | Nitrogen | Ammonia | Ammonia is a chemical compound whose molecule is formed by one nitrogen atom and three hydrogen atoms. |
| 7 | Nitrogen | Fertilizer | Fertilizers used in agriculture provide nitrogen by including urea, ammonium nitrate or ammonium sulfate in their composition. |
| 8 | Oxygen | Water bottle | Water is a chemical compound whose molecule is formed by two hydrogen atoms and one oxygen atom. |
| 9 | Fluorine | Toothpaste | Toothpaste contains fluoride and sodium in the form of sodium fluoride to strengthen enamel. |
| 10 | Neon | Car headlight | The gas in a car's headlights is neon. |
| 11 | Sodium | Bleach | Bleach is a solution containing chlorine and sodium in the form of sodium hypochlorite. |
| 11 | Sodium | Toothpaste | Toothpaste contains fluoride and sodium in the form of sodium fluoride to strengthen enamel. |
| 11 | Sodium | Salt | Table salt, or sodium chloride, is a chemical compound consisting of sodium and chlorine. |
| 12 | Magnesium | Racing bikes | The frames of some racing bikes are made of magnesium alloys. |
| 13 | Aluminium | Italian coffee maker | Italian coffee makers are made of aluminium alloys. |
| 13 | Aluminium | Window frame | A window frame is made of aluminium alloy. |
| 13 | Aluminium | Rocket | Some components in the aerospace industry are made of aluminium and scandium alloys. |
| 13 | Aluminium | Fork | Forks are usually made of aluminium. |
| 14 | Silicon | Window glass | Window glass includes silicon dioxide among its components. |
| 14 | Silicon | Camera lens | The lens of a camera contains silicon. |
| 14 | Silicon | Credit card | The microchip in credit cards is made of antimony on crystalline silicon. |
| 15 | Phosphorus | Matches | Matches contain phosphorus. |
| 16 | Sulfur | Anti-dandruff shampoo | Anti-dandruff shampoo contains selenium disulphide, combining sulfur for its purifying benefits with selenium for its antifungal properties. |
| 17 | Chlorine | Bleach | Bleach is a solution containing chlorine and sodium in the form of sodium hypochlorite. |
| 17 | Chlorine | Salt | Table salt or sodium chloride is a chemical compound consisting of sodium and chlorine. |
| 18 | Argon | Fluorescent lamp | The gas contained in an energy-saving fluorescent lamp is a mixture of argon and krypton, which reduces energy consumption. |
| 19 | Potassium | Banana | Bananas are rich in potassium. |
| 20 | Calcium | Milk | Milk is a good source of calcium. |
| 21 | Scandium | Rocket | Some components in the aerospace industry are made of aluminium and scandium alloys. |
| 22 | Titanium | Supermagnetic magnet | Some types of magnets are made of niobium and titanium alloys. |
| 22 | Titanium | Nail polish | Nail polishes are made of titanium dioxide. |
| 22 | Titanium | Painting | Paints use titanium dioxide as a white pigment. |
| 23 | Vanadium | Adjustable wrenches | Adjustable wrenches often contain vanadium in their steel to enhance strength and durability." |
| 24 | Chrome | Audio tape | A magnetic audio tape consists of a layer of chromium dioxide on a layer of diiron trioxide. |
| 25 | Manganese | Safe deposit box | Safes are made of a manganese steel alloy. |
| 26 | Iron | Audio tape | A magnetic audio tape consists of a layer of chromium dioxide on a layer of diiron trioxide. |
| 27 | Cobalt | Razor blade | Some razor blades are made of cobalt steel alloys. |
| 28 | Nickel | Euro coin | €1 and €2 coins are made from metal alloys that include nickel, such as cupronickel and nickel brass, to enhance durability. |
| 29 | Copper | Solar panel | Some solar panels are made using copper indium selenide, a material used in thin-film photovoltaic technology. |
| 30 | Zinc | Anti-corrosion coating | Some materials are coated with zinc as a protective layer against steel corrosion. |
| 31 | Gallium | Computer memory | Many computer components are made of arsenic and gallium in the form of gallium arsenide. |
| 32 | Germanium | Night vision goggles | The lenses of night vision or infrared goggles are made of germanium. |
| 33 | Arsenic | Computer memory | Many computer components are made of arsenic and gallium in the form of gallium arsenide. |
| 33 | Arsenic | Buckshot | Pellets are small lead spheres, to which arsenic is added to harden the lead. |
| 34 | Selenium | Anti-dandruff shampoo | Anti-dandruff shampoo contains selenium disulfide, combining sulfur for its purifying benefits with selenium for its antifungal properties. |
| 35 | Bromine | Photographic film | Photographic films contain bromine and silver as they are made by a layer of silver bromide on cellulose acetate. |
| 36 | Krypton | Fluorescent lamp | The gas contained in an energy-saving fluorescent lamp is a mixture of argon and krypton, which reduces energy consumption. |
| 37 | Rubidium | Purple fireworks | The purple color of fireworks is due to rubidium present as rubidium salts. |
| 38 | Strontium | Red fireworks | The red color of fireworks is due to strontium in the form of strontium nitrate or strontium carbonate. |
| 39 | Yttrium | Television screens | Yttrium oxide is used in television screens to generate red light in color displays. |
| 40 | Zirconium | Capsule for percussion instrument | The capsules of some percussion musical instruments are made of zirconium alloys. |
| 41 | Niobium | Supermagnetic magnet | Some types of magnets are made of niobium and titanium alloys. |
| 42 | Molybdenum | Lubricant | Some lubricants are made of molybdenum disulfide. |
| 43 | Technetium | Test tube with radioactive material | The test tube stored in the medical coat contains technetium, a radioisotope used in nuclear medicine for disease diagnosis. |
| 44 | Ruthenium | Fountain pen nib | Some ruthenium and osmium compounds are used to manufacture fountain pens. |
| 45 | Rhodium | Exhaust pipe | The catalytic converters used by automobiles in the exhaust pipe to reduce pollution from combustion gases are made of rhodium, platinum or palladium. |
| 46 | Palladium | Exhaust pipe | The catalytic converters used by automobiles in the exhaust pipe to reduce pollution from combustion gases are made of rhodium, platinum or palladium. |
| 47 | Silver | Candlestick | The candlesticks are made of silver. |
| 47 | Silver | Photographic film | Photographic films contain bromine and silver as they are made by a layer of silver bromide on cellulose acetate. |
| 48 | Cadmium | Screw | Some screws are made of cadmium alloys. |
| 49 | Indium | Solar panel | Some solar panels are made using copper indium selenide, a material used in thin-film photovoltaic technology. |
| 50 | Tin | Canned food can | The tinplate of canned food containers has a tin coating to protect it. |
| 51 | Antimony | Credit card | The microchip in credit cards is made of antimony on crystalline silicon. |
| 52 | Tellurium | Rubber | The vulcanization of car tire rubber uses tellurium. |
| 53 | Iodine | Shrimp | Shrimp are rich in iodine. |
| 53 | Iodine | Halogen lamp | Halogen lamps contains iodine. |
| 54 | Xenon | Projector | Xenon gas is used in projection lamps as a light source. |
| 55 | Cesium | Elevator | Infrared sensors in elevator doors use cesium nitrate. |
| 56 | Barium | Rat poison | Rat poison includes a chemical compound called barium carbonate. |
| 57 | Lanthanum | Lighter flints | Lighter flints are made of ferrocerium alloy, which contains cerium and lanthanum as the main elements. |
| 72 | Hafnium | Nuclear submarine | Some nuclear submarines use hafnium in the nuclear reactor control rods. |
| 73 | Tantalum | Mobile phones | Mobile phones contain tantalum, mainly in the form of tantalum capacitors used for efficient energy storage. |
| 74 | Tungsten | Light bulb filament | The filament of an incandescent bulb is made of tungsten. |
| 74 | Tungsten | Gas kitchen | The thermocouple used by a gas kitchen oven to measure temperature is made of tungsten and rhenium. |
| 75 | Rhenium | Gas kitchen | The thermocouple used by a gas kitchen oven to measure temperature is made of tungsten and rhenium. |
| 76 | Osmium | Fountain pen nib | Some ruthenium and osmium compounds are used to manufacture fountain pens. |
| 77 | Iridium | Spark plug | Car spark plugs are made of an iridium alloy to facilitate cold ignition. |
| 78 | Platinum | Exhaust pipe | The catalytic converters used by automobiles in the exhaust pipe to reduce pollution from combustion gases are made of rhodium, platinum or palladium. |
| 79 | Gold | Medal | The medals awarded at the Olympics are often made of gold. |
| 80 | Mercury | Thermometer | Several decades ago, thermometers used mercury as a liquid that expands or contracts with temperature changes. They are no longer sold because of their toxicity. |
| 81 | Thallium | Insecticide | Some insecticides contain thallium, but its use has been  discontinued in some countries due to its high toxicity. |
| 82 | Lead | Car battery | A car battery is made up of a container with several lead plates submerged in sulfuric acid. |
| 82 | Lead | Buckshot | Pellets are small lead spheres, to which arsenic is added to harden the lead. |
| 83 | Bismuth | Fire extinguisher | Fire extinguishers are made from alloys containing bismuth. |
| 92 | Uranium | Nuclear fuel | Nuclear power plants use uranium and plutonium as fuel. |
| 94 | Plutonium | Nuclear fuel | Nuclear power plants use uranium and plutonium as fuel. |
