# Supplementary material for: Elemental Home: A Video Game to Explore Chemistry in Everyday Life
Source: J Chem Educ. 2025 Aug 4;102(8):3716–24. doi: 10.1021/acs.jchemed.5c00168 (PMC12355905; doi:10.1021/acs.jchemed.5c00168)
Supplement: Supplementary file 4 [file ed5c00168_si_004.docx]

**SUPPORTING INFORMATION B**

***Elemental Home*: A video game to explore chemistry in everyday life**

Pedro Juárez-González*, María José Cano-Iglesias, Daniel Cebrián-Robles, and Antonio Joaquín Franco-Mariscal

Universidad de Málaga, Science Education, 29010, Málaga, Spain. Email: [pedrojg94@uma.es](mailto:pedrojg94@uma.es)

**User manual for how to download, install, and play the video game**

**1.** Visit the ENCIC Games website: <https://encic.itch.io>. There, you’ll find a variety of educational games developed by the ENCIC research group.

**2.** Select “**Elemental Home”**. You will see two options:

- **Run game (Play online):** Launch the game directly in your browser (no download needed).
- **Download:** Choose the version for PC or Android.


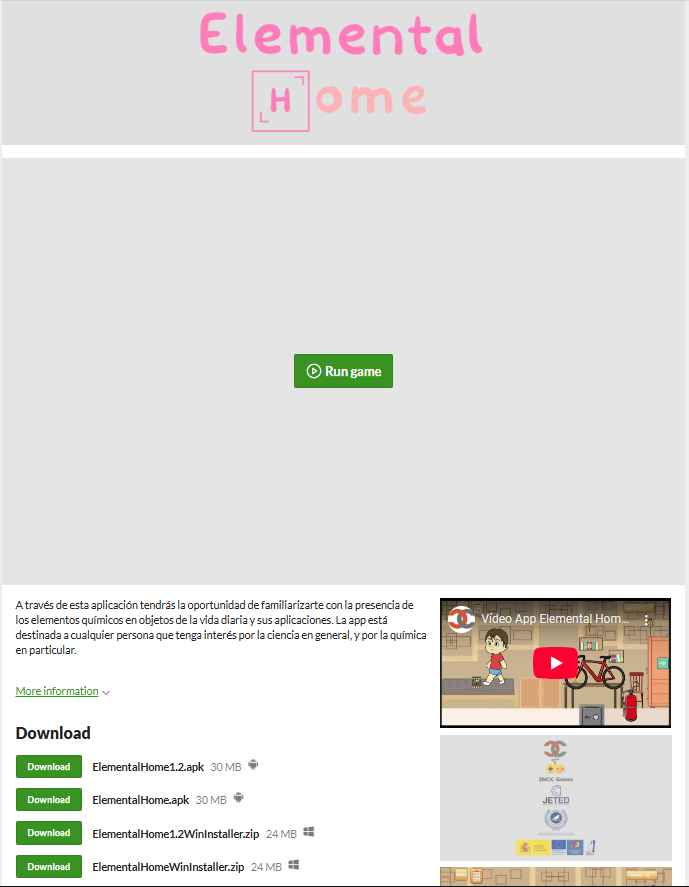


**3. Download and installation:**

- **Version for PC:** (1) Download the latest version of the installation file “ElementalHome(version)WinInstaller.zip”. (2) Follow the setup instructions. (3) Once installed, launch the game from your desktop or start menu.
- **Version for Android:** (1) Download the latest version of the installation file “ElementalHome(version).apk”. (2) If prompted, enable installation from unknown sources in your device settings. (3) Complete the installation and launch the game.

**4.** When you launch the video game for the first time, create a user account by entering a nickname, email, and password. You must accept the privacy policy to proceed. Choose your language (English or Spanish) and avatar (boy, non-binary or girl). Finally, click the "Submit" button to start the video game.


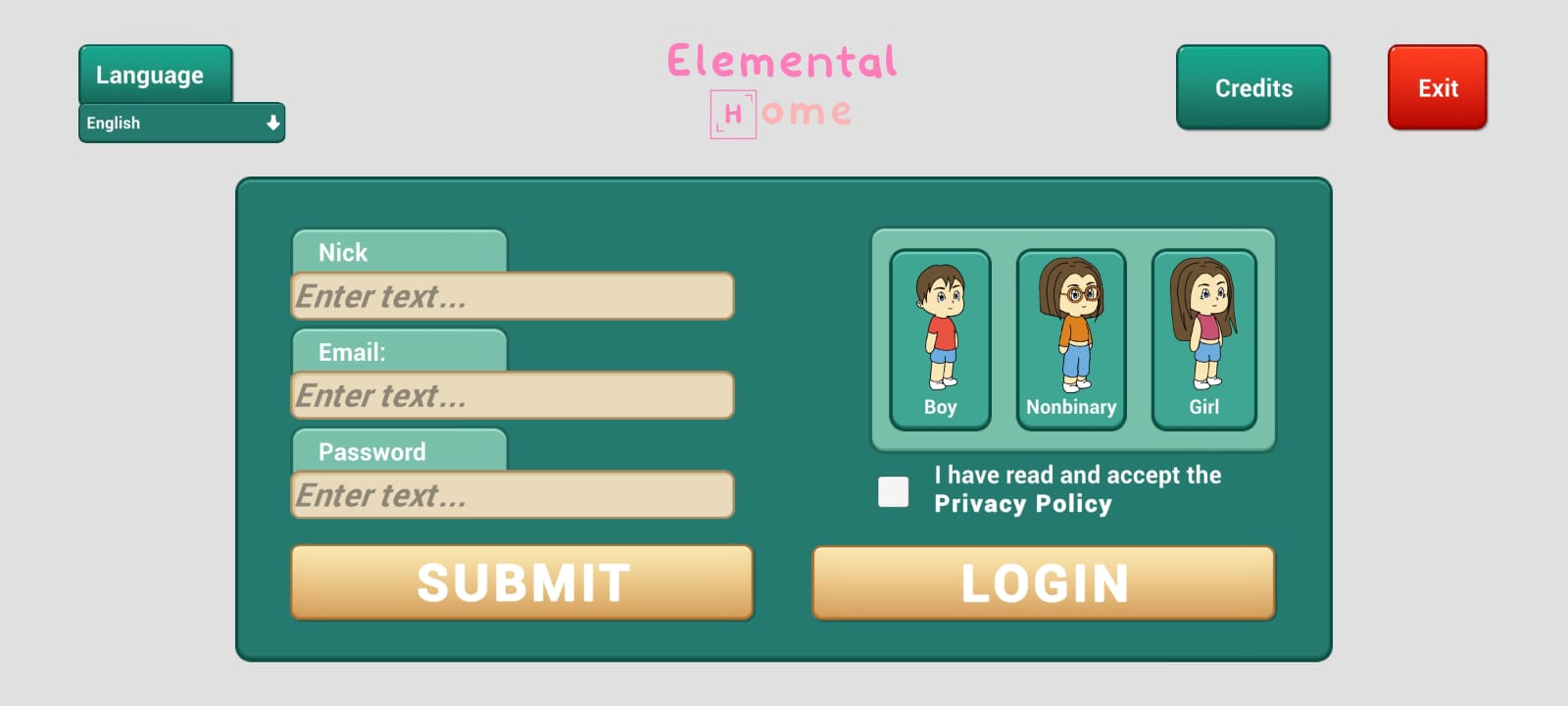


**5.** To play future sessions, click the "Login" button to enter the game.

6. The video game begins with a short quiz where players identify chemical elements and their presence in everyday objects.

7. The video game gives the following explanation on how to play:

*“Hello! We are a group of scientists who are conducting research on chemical elements and their applications. We need your help to obtain several objects from this house where certain chemical elements we want to analyze in our laboratory are present.*

*Remember that chemical elements can be present in objects in various ways:*

*(a) Elemental substance: Elements can exist as pure substances. For example, carbon in the graphite core of a pencil.*

*(b) Chemical compound: elements can combine with each other to form chemical compounds. For example, water (H_2_O) is a compound that contains the elements hydrogen and oxygen. The properties of compounds are different from those of the elements that make them up.*

*(c) States of matter: elements and compounds can exist in solid, liquid, and gaseous states.*

*(d) Solutions: elements can also be present in the form of solutions, where they are dissolved in a solvent, typically water. An example is saltwater, which contains the sodium and chloride ions. Solutions are common in food and cleaning products.*

*(e) Alloys: some objects are mixtures of metallic elements. For example, the steel used in a car's structure is an alloy of iron and carbon. Alloys have different properties from individual elements and are used in the manufacturing of objects such as tools, automobiles, etc.*

*Take this list with the chemical elements that we need. You must associate each collected object with a chemical element from the list. Remember that a single object may contain several chemical elements, so choose your association wisely as each object can only be assigned to one element. Click on an item to select it or to receive a hint in exchange for potions. If you want to get more potions, click on the potions icon.*

*Let us know when you are finished. Good luck!”*


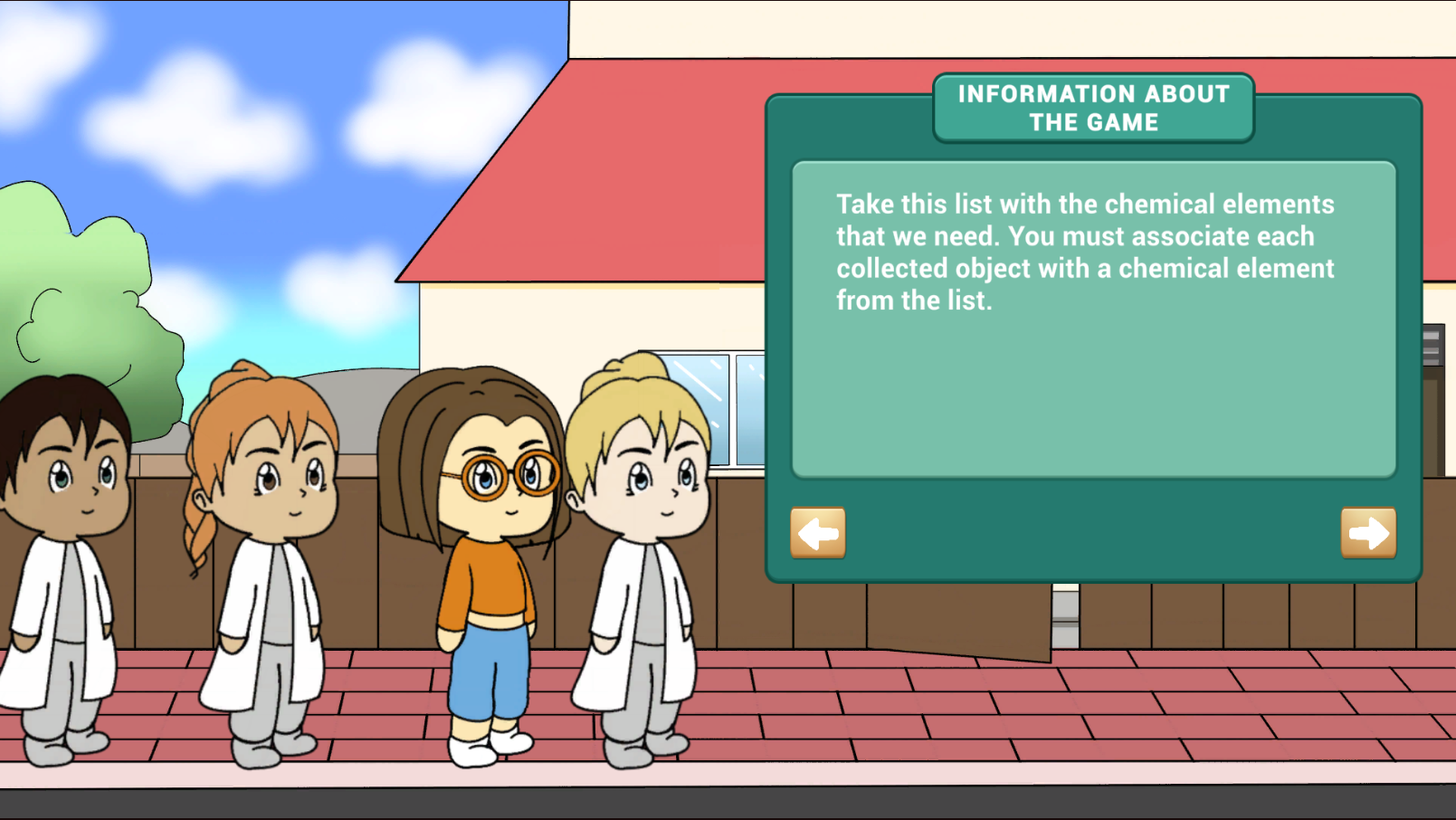


8. When the video game starts, the interface includes the following elements:

- In the top-left corner, you'll find the menu and the list of chemical elements to locate around the house.
- In the top-right corner, you'll see potions, which you can use to request hints. Click the potion icon to answer a question and earn more potions.

9. **By clicking on an object or material in the house, a screen will appear with two options: “Assign element” or “Ask for a hint” about the element.**


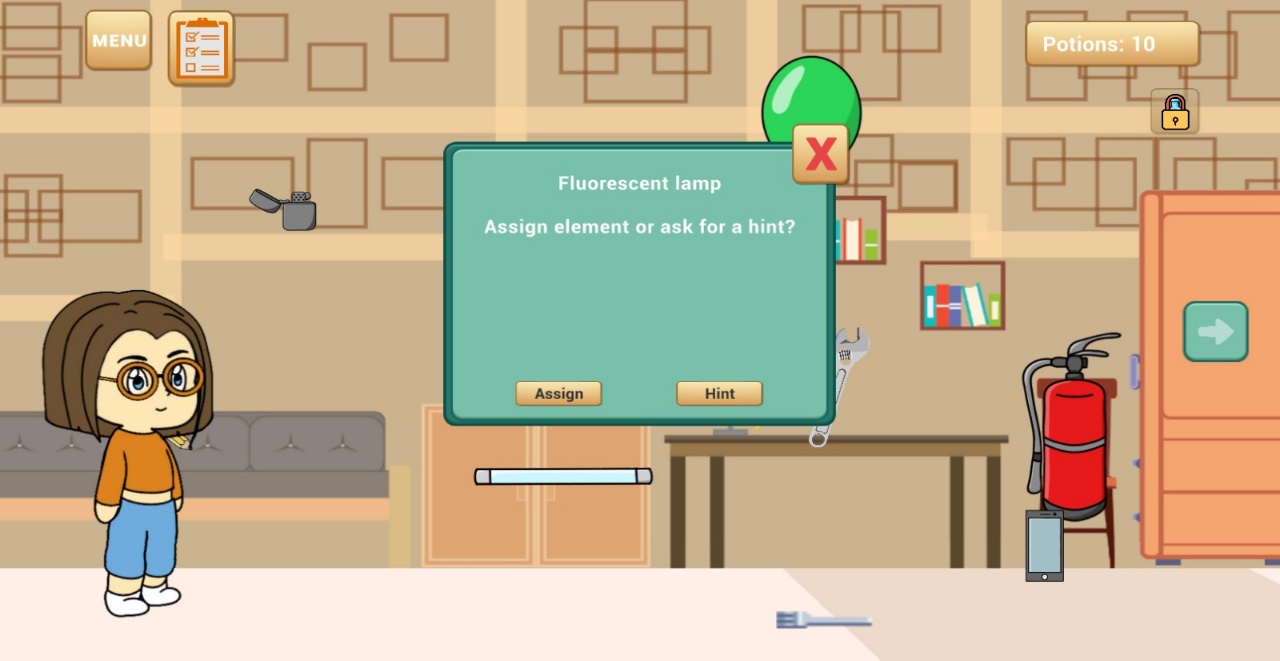


- **Assign element: If you choose this option, a menu will open allowing you to link the object to a chemical element. You** can later modify the assignment of an element by selecting a different associated object.


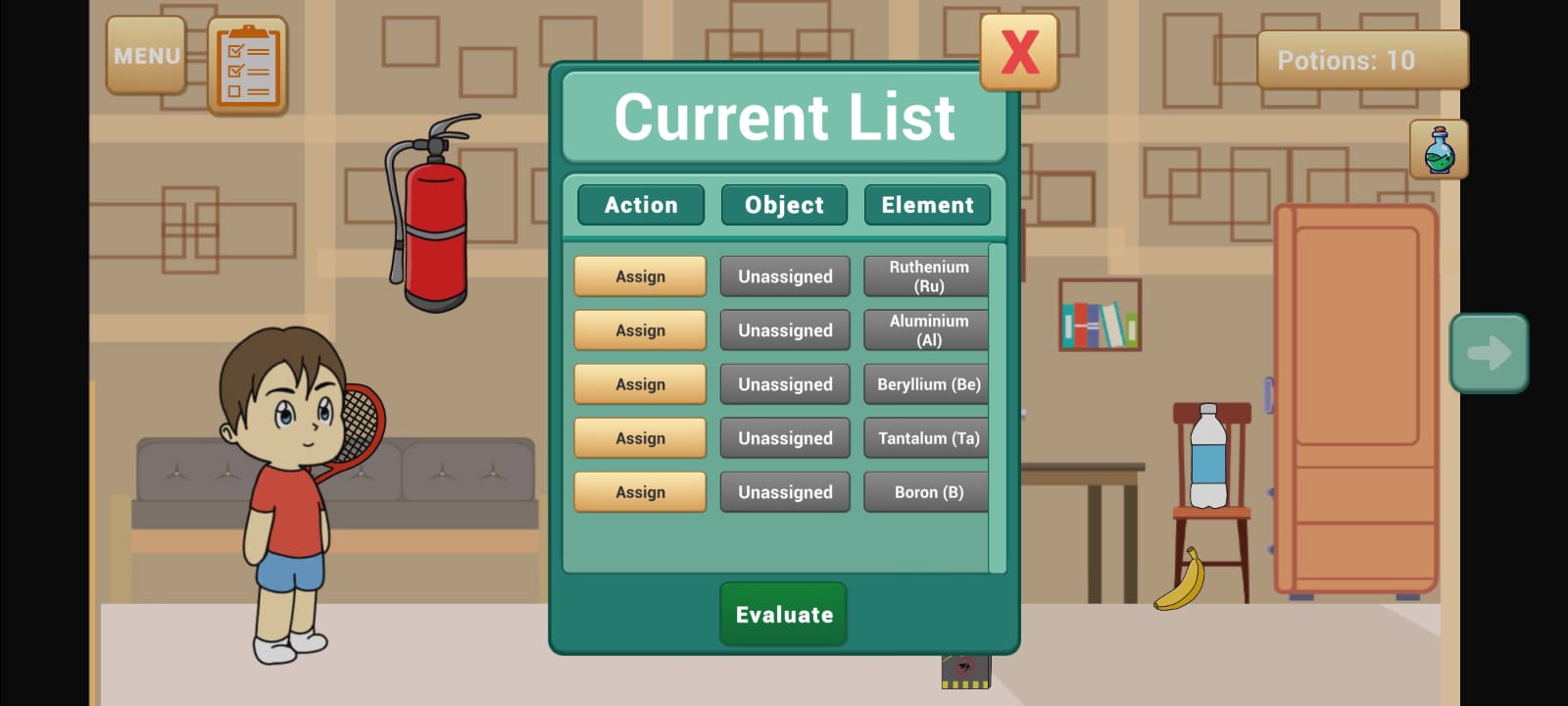


- **Ask for a hint: This option provides information about the element but consumes two potions each time it is used.**


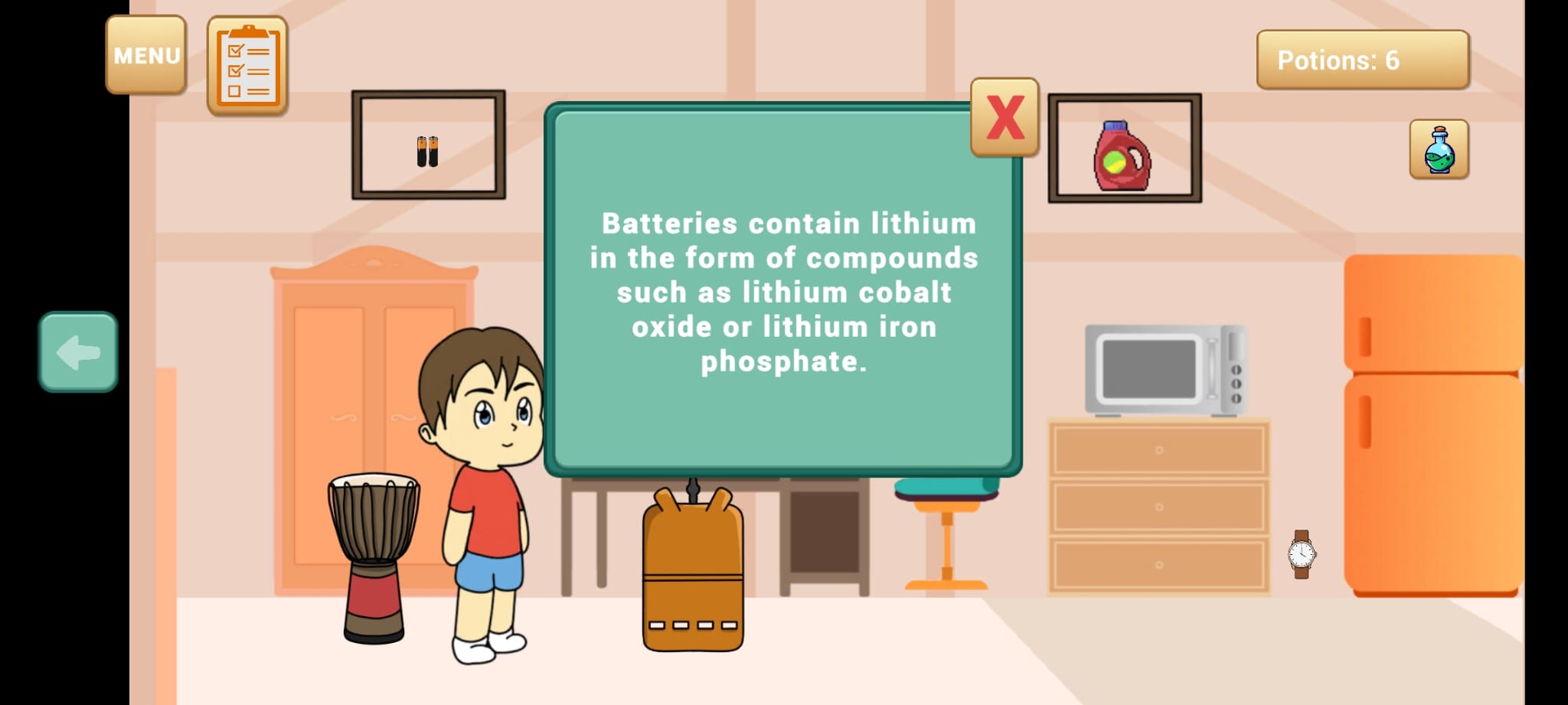


9. After assigning an object to each element on the list, click the “Evaluate” button to submit your answers.

10. The video game will assess your performance. If you pass the level, your score and completion time will be added to the leaderboard, which displays the top five fastest players.

11. Completing a level automatically unlocks the next difficulty level.
